# Novel insights into transmission routes of *Mycobacterium avium* in pigs and possible implications for human health

**DOI:** 10.1186/1297-9716-45-46

**Published:** 2014-04-17

**Authors:** Angelika Agdestein, Ingrid Olsen, Anne Jørgensen, Berit Djønne, Tone B Johansen

**Affiliations:** 1 Norwegian Veterinary Institute, P.O. Box 750 Sentrum, N-0106 Oslo, Norway; 2 Norwegian Pig Health Service, Animalia, P.O. Box 396 Økern, N-0513 Oslo, Norway

## Abstract

*Mycobacterium avium* infection is a severe condition in humans, whereas pigs are often subclinically infected. Pig carcasses represent a possible source of human infection. Faecal excretion of *M. avium* was recently demonstrated in experimentally infected pigs, along with detection of *M. avium* in apparently normal lymph nodes. The present study investigates faecal excretion in naturally infected herds and the presence of live mycobacteria in lymph nodes. Two pig herds (A and B), with a history of sporadically suspected *M. avium* infection were sampled. Herd B used peat, as opposed to Herd A. Samples from peat, sawdust, drinking water, faeces and lymph nodes were collected. Identification of mycobacteria was performed by 16S rDNA sequencing and PCR. *Mycobacterium avium* isolates were analysed by Multi-Locus Variable Number of Tandem repeat Analysis (MLVA). *Mycobacterium avium* subsp. *hominissuis* was detected in samples of faeces, peat and lymph nodes from Herd B, often with identical MLVA profiles. Additionally, other non-tuberculous mycobacteria (NTM) were found in the same material. The absence of macroscopic lymph node lesions in the presence of *M. avium* subsp. *hominissuis* was frequently demonstrated. In Herd A, only one NTM isolate, which proved not to be *M. avium*, was found. Faeces might facilitate transmission of *M. avium* subsp*. hominissuis* between pigs and maintain the infection pressure in herds. The low incidence of macroscopic lesions together with the massive presence of *M. avium* subsp. *hominissuis* in lymph nodes from pigs kept on peat raises questions related to animal husbandry, food safety and human health.

## Introduction

Non-tuberculous mycobacteria (NTM) comprise a wide group of bacteria, where many can act as opportunistic pathogens [1]. *Mycobacterium avium* subsp. *hominissuis* is the most commonly detected NTM causing infection in humans and pigs [2]. *Mycobacterium avium* subsp. *avium* occasionally infects pigs and humans, but is generally regarded as an obligate pathogen of birds, causing contagious avian tuberculosis [3]. Only *M. avium* subsp. *hominissuis* has been detected as the cause of porcine and human cases of *M. avium* infection in Norway [4]. However, data is limited in this regard. Less frequently, other NTM species are described in association with porcine lymphadenitis [5], and some of these have also been described as causative agents in human disease [6].

In humans, pulmonary or systemic manifestations of *M. avium* infection are severe and mainly affect immunocompromised patients and patients with underlying chronic lung conditions [1]. These cases require long-term multi-drug treatment, with antibiotic resistance being a complicating factor. Another typical manifestation of *M. avium* infection is lymphadenitis in the head-and-neck region of children [7], a condition to be treated by surgical intervention. In pigs, no predisposition to severe clinical manifestations of *M. avium* infection has been described. Outbreaks of *M. avium* infection with marked clinical illness are relatively rare in pigs, but in the sporadically reported cases the infection seems to cause reproductive disorders [8,9]. The most common manifestation of infection in pigs, however, is characterised by sub-clinical infection of the lymph nodes and lymphatic tissues of the digestive tract, including the mandibular lymph nodes [10,11]. Dissemination to liver and lungs may also be detected at slaughter without any preceding pronounced clinical symptoms [12]. Inspection and incision of mandibular lymph nodes has been the routine examination for mycobacterial infection at abattoirs, but is no longer practiced in all Norwegian abattoirs. Infection with mycobacteria typically induces formation of granulomatous lesions [13], and the detection of these might cause partial or total condemnation of pig carcasses at the abattoir and lead to financial losses for the farmer.

Cases of human infection with *M. avium* have typically been traced to drinking water systems, saunas, pools and organic environmental materials [14-17]. However, studies demonstrating *M. avium* subsp. *hominissuis* in muscle tissue of experimentally infected pigs [18,19] and in lymph nodes without visible lesions at slaughter [20,21], together with the close genetic relationship between isolates from humans and pigs [4,22-25], make it difficult to rule out the zoonotic potential of *M. avium*.

The most frequently used bedding materials in the swine industry are sawdust and peat. Peat has become increasingly popular in the swine industry the last years, both as an iron enriched feed supplement and as bedding. As bedding, peat has many advantages, like regulation of intestinal function, absorption of moisture and supporting the pigs’ natural behavior. However, both peat and sawdust are well known sources for *M. avium* infection [20,26,27]. Czech screening studies of environmental samples from pig herds also detected *M. avium* subsp. *hominissuis* in water, pig faeces, and invertebrates [28]. A recent experimental infection study in Norwegian pigs compared one clinical *M. avium* subsp. *hominissuis* isolate from a pig and one clinical *M. avium* subsp. *avium* isolate from a bird. The study demonstrated faecal excretion of the *M. avium* subsp. *hominissuis* isolate exclusively [21]. The pronounced shedding was detected six weeks after infection, at about 12 weeks of age. However, in the same study, both subspecies showed similar potentials of establishing infection in pigs.

To the author’s best knowledge, no studies have yet explored the role of faecal excretion of *M. avium* on transmission between pigs in problem herds. The aim of the present study was to investigate the presence of *M. avium* in herds with a history of sporadic cassations due to mycobacteriosis, by examining faeces and lymph nodes of the same group of animals, as well as environmental sources, and by characterising the genetic relationship between the isolates. Additionally, the study aims at assessing the presence of *M. avium* in macroscopically unapparent lymph nodes in natural infected herds, and to hereby elucidate aspects of food safety.

The presence of *M. avium* subsp. *hominissuis* was confirmed in one of the two herds, both in faeces, lymph nodes and in peat, in addition to other NTM species. Multi-Locus Variable Number of Tandem repeat Analysis (MLVA) analysis of a selection of the *M. avium* subsp*. hominissuis* isolates identified 13 different profiles and demonstrated clustering of isolates from peat, faeces and lymph nodes. Also, the presence of *M. avium* subsp. *hominissuis* and other NTM was frequently demonstrated in a high proportion of lymph nodes of normal appearance at meat inspection. In contrast, *M. avium* was not detected in any of the samples from the other herd, and only one NTM isolate was detected in a faecal sample. This herd did not use peat as bedding for the animals.

## Materials and methods

### Herds and animals

Two commercial pig herds practicing combined breeding and fattening of grower pigs, further referred to as Herd A and B, were selected as the study population. Both herds were situated in south-eastern Norway, and had previous reported condemnation of carcasses at slaughter, due to macroscopic changes suggestive of mycobacterial infection in lymph nodes or other internal organs. In Herd A, *M. avium* infection had sporadically been confirmed by culture of tissue samples with lesions during the preceding three years. No such confirmation had been done in Herd B.

Herd A is an integrated farm with 135 breeding sows, weaning about 3500 piglets per year and producing about 2000 slaughter pigs per year. All pigs are kept indoors. Sows with piglets and weaned piglets are kept in different rooms. Dry sows are loose on sawdust bedding. Slaughter pigs are kept in ordinary pens in another house. Herd B is an integrated farm with 68 breeding sows, weaning about 1500 piglets per year and producing approximately 800 slaughter pigs per year. All pigs are kept indoors. Sows with piglets and weaned piglets are kept in the same room. Dry sows are loose on deep straw bedding and slaughter pigs are kept in ordinary pens. In Herd A, sawdust was used as bedding while in Herd B, peat and sawdust were routinely used as bedding for piglets and weaned pigs up to the age of nine weeks. Water was provided from the public drinking water system in both herds, and the animals were fed commercially produced feed (Felleskjøpet®, Oslo, Norway), which had been heat-treated and not added any meat products. No interventions were done concerning the regular everyday animal husbandry or feeding in the herds within the sampling period.

### Sampling

Environmental samples were collected from sawdust, peat and drinking water used at the facilities (Table 1). Sampling of sawdust and peat was performed in storage rooms by random collection of 20 × 10 g of each type of material, whereas one sample of 10 L of drinking water was collected directly from an indoor water tap within each facility.

**Table 1:** Number of samples from pigs and their environment examined for mycobacteria in two commercial herds

**Sample type**	**Herd A**	**Herd B**
Peat	na	20
Water	1	1
Sawdust	20	20
Faeces from sows	15	17
Faeces at 6 weeks of age	95	146
Faeces at 8 weeks of age	95	164
Mandibular lymph nodes	95	123
Jejunal lymph nodes	nd	127

na = not applicable, nd = not done.

Individual faecal samples were collected rectally from the pigs at the age of six and eight weeks and from their mother sows. In Herd A, 95 grower pigs and 15 sows were sampled, and in Herd B, 146 and 164 pigs were sampled at weeks six and eight respectively, in addition to 17 sows (Table 1). One mandibular lymph node was collected from each animal at conventional slaughter (Table 1). Slaughter of the animals from Herd A was concentrated on the same day at the age of approximately 23 weeks, whereas the animals in Herd B were slaughtered in groups on a weekly basis, at the age of approximately 24, 25, 26 and 28 weeks. In addition to the mandibular lymph nodes, one jejunal lymph node per animal was sampled from Herd B (Table 1). The selection of the jejunal lymph node samples was performed randomly in each animal, unless abnormal findings in consistency, size or colour were observed. Such altered lymph nodes were deliberately prioritized for sampling.

### Culturing, isolation and identification of mycobacteria

Lymph nodes were incised and inspected prior to processing in the laboratory, and the presence of macroscopic lesions compatible with caseous necrosis and/or calcification was recorded. All samples were decontaminated as previously described [20] and cultured on slants of Middlebrook 7H10 (BD Diagnostics, Sparks, MD, USA) w/10% oleic acid (BD Diagnostics), with and without antibiotics and fungicides (final concentrations of 100 μg/mL carbenicillin, 200 U/mL polymyxin B sulphate, 19.5 μg/mL trimethoprim lactate and 10 μg/mL amphotericin B), Dubos PS [29] and Stonebrink’s medium (BD Diagnostics). Slants were incubated for eight weeks at 37 °C, and colonies resembling mycobacteria were sub-cultured on Middlebrook 7H10 and the medium they were initially observed to grow on. On primary isolation, attempt was made to pick one colony of each morphotype, when more than one was present.

Isolates shown to be acid-fast rods by the Ziehl-Neelsen (ZN) staining method were further examined by PCR targeting the *M. avium* specific IS element IS*1245* (primers p40 and p41) and the *M. avium* subsp. *avium* specific IS*901* (primers p901a and p901c) as previously described [30,31]. Isolates negative on the PCR reactions were analysed further by 16S rDNA sequencing of the 151 bp hypervariable region A to identify species, as described [32]. Primers are presented in Additional file 1. Sequencing reactions were run partly in-house and partly by GATC Biotech (Konstanz, Germany), and the obtained sequences were analysed by using the Basic Local Alignment Search Tool (BLAST) [33] (National Center for Biotechnology Information (NCBI), Bethesda, MD). Maximum scores and maximum identity of ≥ 99% were accepted.

### Investigation of relatedness between *M. avium* isolates

Isolates of *M. avium* were analysed by Multi-Locus Variable Number of Tandem repeat Analysis (MLVA) of 8 loci, as previously described [34]. Loci and primers are presented in Additional file 1. The PCR was performed using HotStar Taq® polymerase (Qiagen, Hilden, Germany) at the following thermocycling conditions: Initially 95 °C for 15 min, then 40 cycles of 30 s at 95 °C, 30 s at 58 °C, and 30 s at 72 °C, and finally 1 cycle of 7 min at 72 °C. The reference strain *M. avium* subsp. *avium* ATCC 25291 was used as positive control in each run. Sizes of the PCR products, reflecting differences in repeat numbers, were determined by using the Agilent Bioanalyzer® (Agilent Technologies, Santa Clara, CA, USA) and converted to a corresponding number for each locus as described by Thibault et al. [34]. Cluster analysis was performed using the categorical method and the unrooted UPMGA tree in Bionumerics 6.1 (Applied Maths, Sint-Martens-Latem, Belgium). Only isolates of 100% similarity, i.e. isolates having the same number of tandem repeat in each locus, were assigned to the same cluster.

## Results

### Culturing, isolation and identification of mycobacteria

No mycobacteria were detected in samples of sawdust and water from either of the two herds.

In Herd A, only one mycobacterial isolate was detected. It was found in a faecal sample from a six week old pig, and identified as *M. senuense*. Neither macroscopic lesions nor mycobacteria were detected in mandibular lymph nodes from Herd A.

In Herd B, mycobacterial species were detected in peat, and in faeces from sows and piglets. Nineteen samples of peat (95%), three samples of sow faeces (18%), and 44 faecal samples (14%) from six and eight week old pigs contained mycobacteria. *Mycobacterium avium* subsp. *hominissuis* was detected from 11 samples of peat (55%) and from seven faecal samples (2%) from six and eight week old pigs, while *M. avium* was not detected in sow faeces. A total of 16 isolates of *M. avium* subsp. *hominissuis* from the 11 positive peat samples and 11 isolates from the seven positive faecal samples were analysed by MLVA. Other mycobacterial species detected in peat samples were *M. bohemicum* (45% of samples), *M. malmoense* (20%), *M. palustre* (15%) and *M. celatum* (5%). *Mycobacterium malmoense* was identified in faecal samples from two sows, and *M. celatum* from one. In the 310 faecal samples collected from six and eight week old animals, *M. malmoense* (5%), *M. triviale* (5%) *M. bohemicum* (1%), *M. celatum* (1%) and *M. branderi* (0, 5%) were detected. Numbers of isolates of each mycobacterial species within the volume of each sample type are shown in Table 2.

**Table 2:** Number of samples from Herd B where mycobacterial species were detected

**Sample material**	**Peat**	**Faeces**			**Lymph nodes**	
		**Sows**	**16 weeks of age**	**8 weeks of age**	**Mandibular**	**Jejunal**
**Number of samples**	**20**	**17**	**146**	**164**	**123**	**127**
*M. avium *subsp. *hominissuis*	11	-	2	5	81	60
*M. branderi*	-	-	1	-	-	-
*M. bohemicum*	9	-	-	4	8	-
*M. celatum*	1	1	-	2	7	1
*M. malmoense*	4	2	9	8	-	-
*M. palustre*	3	-	-	-	1	-
*M. triviale*	-	-	9	5	-	-
*M.* spp*.* (unidentified)	-	-	-	3	2	-

In the examined mandibular and jejunal lymph nodes from the animals in Herd B, mycobacterial species were detected in 69% and 48%, respectively. *Mycobacterium avium* subsp. *hominissuis* was found in 81 mandibular (66%) and in 60 jejunal lymph nodes (47%) (Table 2). The detection frequency increased with the age of the slaughtered pigs at sampling, developing from 60% at 24 weeks to 80% at 28 weeks in mandibular lymph nodes, and from 33% to 72% in jejunal lymph nodes (Table 3). Other mycobacterial species cultured from the total amount of 250 lymph nodes were *M. bohemicum* (3% of samples), *M. celatum* (3%) and *M. palustre* (0.4%). These NTM were detected alone or together with *M. avium subsp. hominissuis* in the same sample. Also, five additional isolates of acid fast rods remained unidentified, due to the quality of sequences being too poor for 16S rDNA analysis.

**Table 3:** **Detection frequencies of *Mycobacterium avium *** **subspecies *hominissuis *** **in lymph nodes sampled at slaughter from pigs of different weeks of age**

	**Mandibular lymph nodes**			**Jejunal lymph nodes**		
	**Samples examined**	**Positive samples**		**Samples examined**	**Positive samples**	
**Weeks**	**n**	**n**	**%**	**n**	**n**	**%**
**24**	38	23	**60**	33	11	**33**
**25**	45	28	**62**	52	21	**40**
**26**	15	10	**67**	13	7	**54**
**28**	25	20	**80**	29	21	**72**

Grossly detectable changes, such as abnormal consistency, colour or macroscopically visible changes, were recorded in 38 (31%) of the mandibular and 27 (21%) of the jejunal lymph nodes sampled. *Mycobacterium avium* subsp. *hominissuis* was detected in a large number of lymph nodes without lesions, and Table 4 shows the relationship between the presence of grossly detectable changes and the presence of live *M. avium* subsp. *hominissuis*.

**Table 4:** **Detection frequencies of *Mycobacterium avium *** **subspecies *hominissuis *** **in mandibular and jejunal lymph nodes with and without gross pathological lesions from pigs slaughtered at different age**

**Lymph node**	**Weeks**	**Pathological changes present**		**Pathological changes absent**	
**Mandibular**		**Total number**	**Culture positive**	**Total number**	**Culture positive**
	24	8	100%	30	53%
	25	15	93%	30	43%
	26	6	100%	9	44%
	28	10	90%	15	73%
	**Total**	**39**	**95%**	**84**	**52%**
**Jejunal**	**Weeks**	**Pathological changes present**		**Pathological changes absent**	
		**Total number**	**Culture positive**	**Total number**	**Culture positive**
	24	3	100%	30	27%
	25	6	100%	46	33%
	26	3	100%	10	40%
	28	15	100%	14	43%
	**Total**	**27**	**100%**	**109**	**42%**

### Investigation of relatedness between *M. avium* isolates

**Figure 1:**
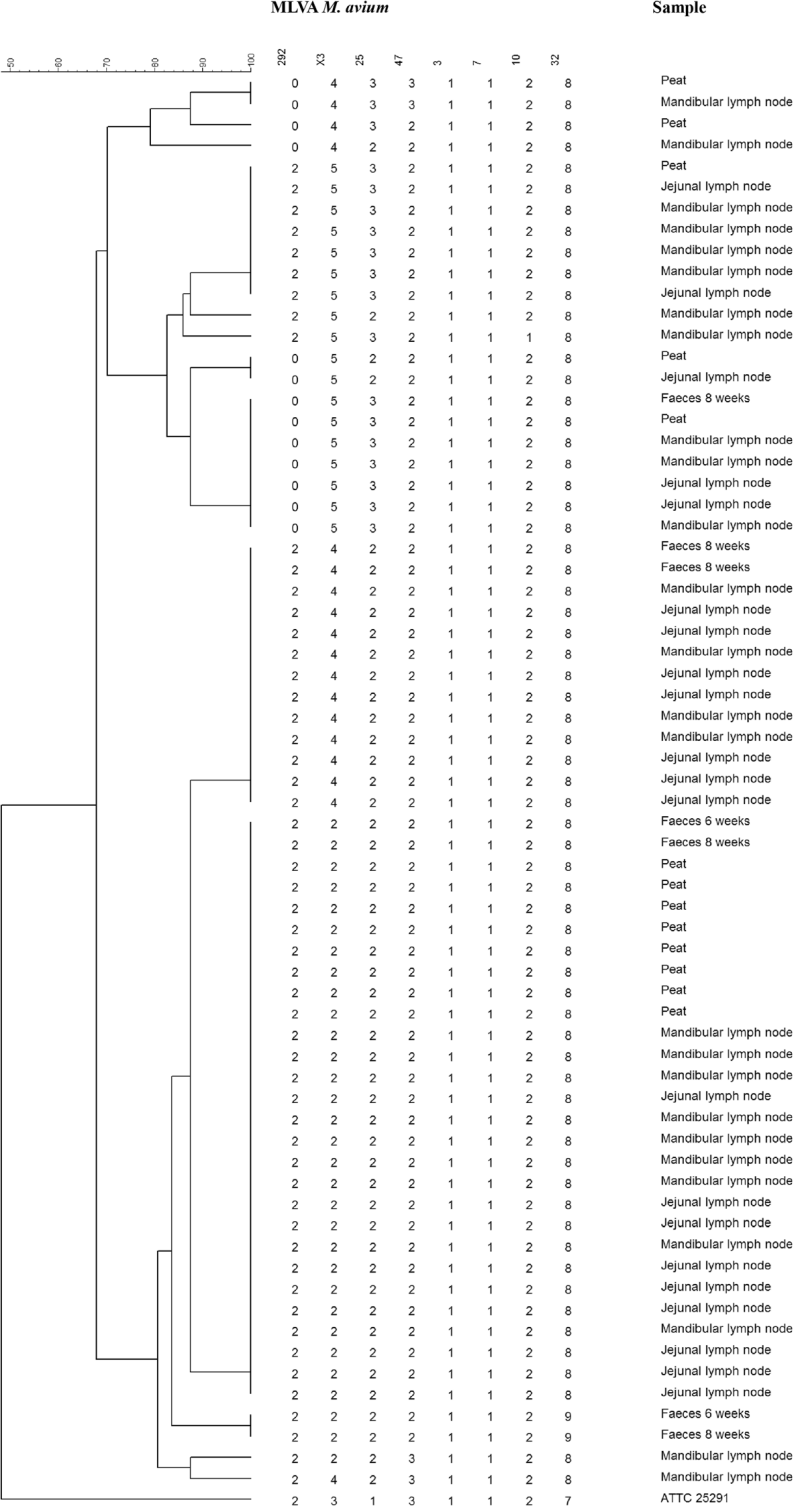
**Dendrogram.** An unrooted tree showing genetic relationship between isolates of *Mycobacterium avium* subsp. *hominissuis* originating from peat, faeces and lymph nodes from slaughtered pigs in a Norwegian herd. The dendrogram is based on eight locus MLVA analysis. *Mycobacterium avium* subsp. *avium* ATTC 25291 is included as reference strain. The tree is created in Bionumerics 6.1, using categorical data and the Unweighted Pair Group Method with Arithmetic Mean (UPGMA).

All isolates from peat (*n* = 16) and faeces (*n* = 11), and a random selection of 47 (33%) of the 141 isolates from lymph nodes, underwent MLVA analysis. MLVA analysis identified 13 different profiles among the 74 analysed isolates, distributed on seven clusters and six singletons (Figure 1). Clusters were recognised when containing ≥ 2 isolates with identical profile. The present analysis showed clusters of different sizes, ranging from two to 28 isolates. In two samples from peat, two different MLVA profiles were detected within the same sample. In the other nine peat and all 11 faecal samples, isolates from the same sample showed identical MLVA profiles, and only one representative from each sample is shown in the dendrogram. Multiple MLVA profiles were detected in all types of sample material, and isolates from peat, faeces and lymph nodes clustered together on several occasions. However, due to the animals not being individually numbered, further investigation of the potential presence of multiple profiles within the same animal was not possible to perform.

## Discussion

The presence of *M. avium* subsp. *hominissuis* in faeces in naturally infected pigs was demonstrated in the present study. Together with the similar finding in our recent study with experimentally infected pigs [21], the suggestion of faeces playing a potential role in the spread of *M. avium* subsp. *hominissuis* infection is strengthened. Our findings are in concordance with older literature on studies of *M. avium* in pigs [35-37], but to our best knowledge, the present study is the first of its kind to methodically study faecal excretion of *M. avium* subsp. *hominissuis* in naturally infected pigs. Additionally, the detection of identical isolates in peat, faeces and lymph nodes, suggests that peat was the primary source of infection for the herd. This assumption is supported by previous research describing a link between *M. avium* infections in swine and peat [20,27]. *Mycobacterium avium* subsp. *hominissuis* in faeces might originate merely from peat passing through the alimentary tract, but the absence of *M. avium* subsp. *hominissuis* in faeces from the sows in the study, who also were kept on peat, support a theory of *M. avium* subsp. *hominissuis* being excreted from jejunal lesions. In our previous study of experimental infection of piglets at an early age with *M. avium* subsp. *hominissuis* and *M. avium* subsp. *avium*, *M. avium* subsp. *hominissuis* was detected in faeces at six weeks, which reinforces the conclusion that the bacterium was not just passing through the digestive system [21]. The frequent detection of *M. avium subsp. hominissuis* in jejunal lymph nodes is also suggestive of a link between intestinal affection and the presence of *M. avium* subsp. *hominissuis* in faeces. A theory of transmission of *M. avium* subsp. *hominissuis* between animals by the faecal-oral route was not rejected by the present MLVA results, regarding the fact that isolates from peat, faeces and lymph nodes clustered together frequently. Faecal excretion of *M. avium* subsp. *hominissuis* might contribute to enhanced pressure of infection in a herd and maintain the possibility of transmission between animals. However, it is not possible to compare the role of faeces and peat in infection of pigs based on the present results.

*Mycobacterium avium* subsp. *hominissuis* was frequently detected in mandibular and jejunal lymph nodes of slaughtered pigs from the breeding facility that used peat, and meat from this herd was used for human consumption without any intervention. Importantly, on several occasions *M. avium* subsp. *hominissuis* was cultured from lymph nodes without any macroscopically visible changes indicative of infection, in concordance with our previous study of experimental *M. avium* infection in pigs [21]. The great extent of *M. avium* subsp. *hominissuis* present within lymph nodes claims for further knowledge about the presence of the bacterium in the remaining porcine organism. The findings suggest that lesions might very well not be visible to the naked eye, and that *M. avium* subsp. *hominissuis* is very likely to be found in carcasses from pigs raised on peat. Similar observations have also been described previously [20,27]. Further investigations could be important in order to secure the health of people handling pork carcasses and meat, and of the consumers. Reports on *M. avium* subsp. *hominissuis* isolates from pigs and humans being closely related [2,4,22-25], together with the fact that a common source of *M. avium* subsp. *hominissuis* infection in these two mammalian species is yet to be identified, strengthen the theory generated by the present study that porcine intestinal organs or pork meat can be a potential source of human *M. avium* subsp. *hominissuis* infection. Other authors have reported detection of *M. avium* subsp. *hominissuis* DNA in muscle tissue of pigs artificially infected with *M. avium* subsp. *hominissuis*[18,19]. The question that remains is whether *M. avium* subsp. *hominissuis* could be present outside of lymph nodes in unapparent porcine carcasses. Further investigations, aiming at thoroughly mapping the presence of *M. avium* subsp. *hominissuis* in muscles, lungs and livers of slaughtered pigs, would be required to secure an up-to-date mode of animal husbandry and meat inspection and thereby protect human health. Depending on the outcome of such future investigations, the need to apply novel techniques at meat control to rapidly identify the presence of *M. avium* in porcine tissues [38] might be appropriate to consider.

In the present study, a variety of other NTM of significance for human health were isolated from peat, faeces and lymph nodes. Although no further attempt was done to investigate the relatedness between isolates to elucidate routes of transmission, the findings could provide helpful information about possible sources for both porcine and human infection. Species isolated from all the three types of sample material were *M. bohemicum*, *M. celatum* and *M. palustre*, of which all have been reported to have relevance for human health. Both *M. bohemicum* and *M. palustre* have been described as causative agents of peadiatric lymphadenitis [39-42] and should be regarded as potential pathogens [6]. *Mycobacterium celatum* can cause disseminated infection or lung infection in humans with underlying disease [6], paediatric lymphadenitis [43] and serious pulmonary infection in apparently immunocompetent individuals [44,45]. Isolation of *M. celatum* from porcine lymph nodes has previously been reported only once [46]. *Mycobacterium malmoense* was found only in peat and faeces in the present study, but has been detected in porcine lymph nodes by others [5]. It is increasingly recognised as an important lung pathogen in humans [47], but has also been associated with human lymphadenitis and septic arthritis [48,49]. *Mycobacterium branderi*, reported to cause human lung disease and ulcerative tenosynovitis [50,51], was on one occasion detected in faeces in the present study. In partially concordance with our present findings, van Ingen et al. [5] cultured *M. malmoense*, *M. bohemicum* and *M. palustre* from porcine lymph nodes. Our supplementary isolation of the same species in peat, together with the absence of NTM in lymph nodes from pigs not raised on peat bedding, suggests peat as a source of infection in pigs. Although the identified NTM species already are considered to be environmental, enrichment in peat and faeces might be of relevance for establishment of infection in pigs, with the further possibility of transmission to humans.

To conclude, faeces might be of importance for transmission of *M. avium* subsp. *hominissuis* between animals and for maintenance of the infection pressure in pig herds. Also, pigs might be heavily infected with *M. avium* subsp. *hominissuis*, despite their carcasses being macroscopically unapparent with regards to mycobacterial presence. Peat represents an important source of *M. avium* subsp. *hominissuis*, but also of other NTM that might be of increasing significance for porcine and human health. All in all, these findings should stimulate further investigation of the presence of mycobacteria in pig carcasses and reconsiderations with regards to animal husbandry, as well as to the current practice of meat control.

## Competing interests

The authors declare that they have no competing interests.

## Authors’ contributions

AA was responsible for conception and design of the experiment, performed sampling, laboratory work, data analysis, and drafted the manuscript. IO contributed to conception and design of the experiment, sampling, and to critical revision of the manuscript. AJ was involved in conception and design of the experiment, sampling and in critical revision of the manuscript. BD participated in conception and design of the experiment, laboratory work and in critical revision of the manuscript. TBJ contributed to conception and design of the experiment, sampling, laboratory work, MLVA data analysis and helped to draft the manuscript. All authors have read and approved the final manuscript.

## Supplementary Material

Additional file 1 **Primers for MLVA analysis and 16 s rDNA sequencing.** Table describing the primers used for the eight locus MLVA analysis and for the 16S rDNA sequencing analysis. Click here for file
