# Supplementary material for: Novel insights into transmission routes of Mycobacterium avium in pigs and possible implications for human health
Source: Vet Res. 2014 Apr 17;45(1):46. doi: 10.1186/1297-9716-45-46 (PMC4021465; doi:10.1186/1297-9716-45-46)
Supplement: Additional file 1 — Primers for MLVA analysis and 16 s rDNA sequencing. Table describing the primers used for the eight locus MLVA analysis and for the 16S rDNA sequencing analysis. [file 1297-9716-45-46-S1.docx]

**Additional file 1 Primers for MLVA analysis and 16s rDNA sequencing.**

|  | **Primers** | |  |
| --- | --- | --- | --- |
| **Locus** | **forward** | **reverse** |  |
| TR3 | 5’-catatctggcatggctccag-3’ | 5’-atcgtgttgaccccaaagaaat-3’ |  |
| TR7 | 5’-gacaacgaaacctacctcgtc-3’ | 5’-gtgagctggcggcctaac-3’ |  |
| TR10 | 5’-gacgagcagctgtccgag-3’ | 5’-gagagcgtggccatcgag-3’ |  |
| TR25 | 5’-gtcaagggatcggcgagg-3’ | 5’-tggacttgagcacggtcat-3’ |  |
| TR32 | 5’-ccacagggtttttggtgaag-3’ | 5’-ggaatccaacagcaaggac-3’ |  |
| TR47 | 5’-cgttgcatttctgcgtagc-3’ | 5’-ggtgatggtcgtggtcatcc-3’ |  |
| TR292 | 5’-cttgagcagctcgtaaagcgt-3’ | 5’-gctgtatgaggaagtctattcatgg-3’ |  |
| X3 | 5’-aacgagaggaagaactaagccg-3’ | 5’-ttacggagcagaaggccagcggg-3’ |  |
| 16S | 5’-agagtttgatc(c/a)tgg(c/t)tcag-3’ | 5’- tttcacgaacgcgacaa-3’ |  |
